# Imagery Rehearsal Based Art Therapy: Treatment of Post-traumatic Nightmares in Art Therapy

**DOI:** 10.3389/fpsyg.2020.628717

**Published:** 2021-01-14

**Authors:** Suzanne Haeyen, Merel Staal

**Affiliations:** ^1^GGNet Centre of Mental Health, Apeldoorn, Netherlands; ^2^Hogeschool van Arnhem en Nijmegen [HAN] University of Applied Sciences, Nijmegen, Netherlands

**Keywords:** post-traumatic stress disorder, art therapy, nightmares, imagery rehearsal therapy, trauma

## Abstract

Imagery Rehearsal Therapy (IRT) is effective for trauma-related nightmares and is also a challenge to patients in finding access to their traumatic memories, because these are saved in non-verbal, visual, or audiovisual language. Art therapy (AT) is an experiential treatment that addresses images rather than words. This study investigates the possibility of an IRT-AT combination. Systematic literature review and field research was conducted, and the integration of theoretical and practice-based knowledge resulted in a framework for Imagery Rehearsal-based Art Therapy (IR-AT). The added value of AT in IRT appears to be more readily gaining access to traumatic experiences, living through feelings, and breaking through avoidance. Exposure and re-scripting take place more indirectly, experientially and sometimes in a playlike manner using art assignments and materials. In the artwork, imagination, play and fantasy offer creative space to stop the vicious circle of nightmares by changing theme, story line, ending, or any part of the dream into a more positive and acceptable one. IR-AT emerges as a promising method for treatment, and could be especially useful for patients who benefit least from verbal exposure techniques. This description of IR-AT offers a base for further research.

## Introduction

Post-Traumatic Stress Disorder (PTSD) is characterized by severe symptoms of re-experiencing, hyper arousal, avoidance, flashbacks, negative beliefs, or expectations, sleep disturbance, and distressing dreams, or nightmares as consequences of one or more traumatizing experiences. It is diagnosed when the duration of the symptoms is more than 1 month and the disturbance causes clinically significant distress or impairment in social, occupational, or other important areas of functioning (American Psychiatric Association, 2013). Events that may lead to PTSD include, but are not limited to, violent personal assaults, natural or human-caused disasters, accidents, combat, and other forms of violence.

Many people who have been diagnosed with a PTSD also have nightmares. In the number of people suffering from PTSD and nightmares is estimated at 40% and as high as 98% (Wittmann et al., 2007; Van Schagen et al., 2012). A common symptom of PTSD are nightmares in which the traumatized person relives his experiences while sleeping (Barlow and Durand, 2015). In addition, people who also suffer from nightmares that do not have a full PTSD diagnosis, but do have post-traumatic symptoms (Van Deth, 2017).

Nightmares are dreams that seem to be real to the dreamer and often lead to feelings of stress and fear. The woken dreamer may initially have trouble comprehending that it was in fact a dream and not real. In the event of PTSD, the nightmare content is directly related to the traumatic event, which is re-experienced during sleep. They are described as replicative nightmares and are considered one of the main PTSD symptoms (American Psychiatric Association, 2013).

Imagery Rehearsal Therapy (IRT) is a Cognitive Behavior Therapy (CBT) technique to inhibit the original nightmare and provide a cognitive shift that empirically refutes the original premise of the nightmare (Lancee et al., 2011; Germain, 2013). Many non-pharmacologic techniques have been proposed to treat PTSD-related or idiopathic nightmares, including hypnosis, lucid dreaming, Eye Movement Desensitization and Reprocessing (EMDR), desensitization, and IRT. However, only desensitization and IRT have been the object of controlled studies, and IRT has received the most empirical support (Krakow and Zadra, 2010). Research has shown effective results of IRT on traumatic nightmares. This method seems to have a beneficial effect on PTSD symptoms, the quality of sleep and the nightmare frequency (Van Schagen et al., 2015; Van Schagen and Lancee, 2018). Nevertheless, there is evidence that not all patients benefit from trauma-oriented cognitive behavioral therapy (Schouten et al., 2018). To tell and expose themselves to their own trauma can be perceived as too great a threshold for these patients, preventing them from accessing the traumatic memories.

Art therapy, as a non-verbal and experiential treatment, consists of and makes use of non-verbal communication of thoughts and feelings, based on the idea that the creative process of art making is healing and life-enhancing. Like other forms of psychotherapy and counseling, it is used to encourage personal growth, increase self-understanding, and assist in emotional repair (Schouten et al., 2018; Malchiodi, 2020). Art therapy differs from other forms of psychotherapy and counseling in its methodical use of techniques such as drawing, painting, collage, and sculpting to shape and express feelings, thoughts, and memories in visual and concrete terms. Art therapy addresses images rather than words, and offers a different kind of access to traumatic memories and emotions than verbal treatments. The non-verbally stored traumatic memory, which is relived during a nightmare, can be examined, shaped in the artwork and thus externalized. In this way, memories can be processed, and negative thoughts and emotions associated with it can be treated (McMillan et al., 2018). Art therapy is visible and tangible through the use of visual material and therefore consistent with coming into contact with the non-verbal, visual, sensory-perceptual nature of traumatic memories (Lobban, 2016; Schouten et al., 2018). In this review we define art therapy as a circumscribed form of psychotherapy grounded in arts-based methodology, such as sensory, movement, visual, and other forms of art-based approaches (Malchiodi, 2020).

Art therapy may offer a treatment alternative for patients who are unable to talk about traumatic memories and unable to tolerate exposure treatments. Art therapeutic interventions provide an opportunity to distance oneself from emotion while offering cognitive integration of emotion and stimulating meaning-making processes (Malchiodi, 2020). Based on expert opinions and preliminary research, a three-phase trauma-focused art therapy protocol has been investigated (Schouten et al., 2014; Elbrecht, 2018). The first phase of the protocol is aimed at stabilization and symptom reduction, the second phase is trauma-focused, and the third phase works on integration and meaning-making. Although the first phase of the art therapy protocol is called stabilization and symptom reduction, its purpose goes beyond stabilization and focuses on decreasing avoidance and accessing traumatic memories as a form of progressive exposure. It is posited that art making in art therapy may provide relaxation and decreased arousal, reducing avoidance by providing more gradual access to traumatic as well as positive memories and emotions, and enabling patients to express and externalize memories and emotions in visual art and to link this to implicit and explicit memory (Malchiodi, 2003, 2012, 2020; Schouten et al., 2014).

Implicit and explicit memory systems help in the understanding of what happens to traumatic memory, and why it is so hard to express traumatic experiences in words. Rotschild (2014) and Levine (2015) have stated on numerous occasions to consider PTSD as a conflict between the ancient brain (implicit memory) and the prefrontal cortex (explicit memory). When humans experience life-threatening situations, most energy is withdrawn from the prefrontal cortex, the area associated with planning, language, logic and interpreting functioning. Instead, the amygdala, as the smoke alarm, activates an instinctual two-path-response in the hippocampus: either active response (running away or fighting) or a passive response (freezing, dissociating). From the moment the hippocampus is activated, large areas of the prefrontal cortex are shut down. The individual is not able to speak or think in a logic way. In this way, the memories of this event will be hold as non-verbal, audio-visual orientated, “frozen” language. An experience what Elbrecht (2018) calls: an unresolved an incomplete response to something that was experienced as an unbearable level of helplessness and being overwhelmed.

When working with explicit memory, words are used to express what we are thinking or feeling and to make sense of our experiences both past and present; we can create stories and put them into perspective. Implicit memory is associated with the subcortical parts of the brain; the primitive parts that are not under conscious control and have no linguistic representation. Implicit memory is created without words, only including sensations and images. Rotschild (2014), Van der Kolk (2014), and Levine (2015) found that the language center shuts down, when experiencing traumatic events. Because there is no verbal or logical processing, the contents of these traumatic memories will be presented in flashbacks and overwhelming images, sounds and emotions. Art therapy might therefore be a good starting point for the treatment of PTSD-related nightmares and for gaining access to traumatic memories. Trauma-focused IRT art therapy emphasizes expression of memories as a way to help individuals expose painful memories and help to make sense of experiences by putting them into (new) narratives (Malchiodi, 2020). Hopeful results were found in a case study by Perryman et al. (2019). The effect of the use of art therapy and the integration of right and left brain, was that the client was able to “feel ‘almost normal’ again and hopeful about life” (Perryman et al., 2019).

**FIGURE 1:**
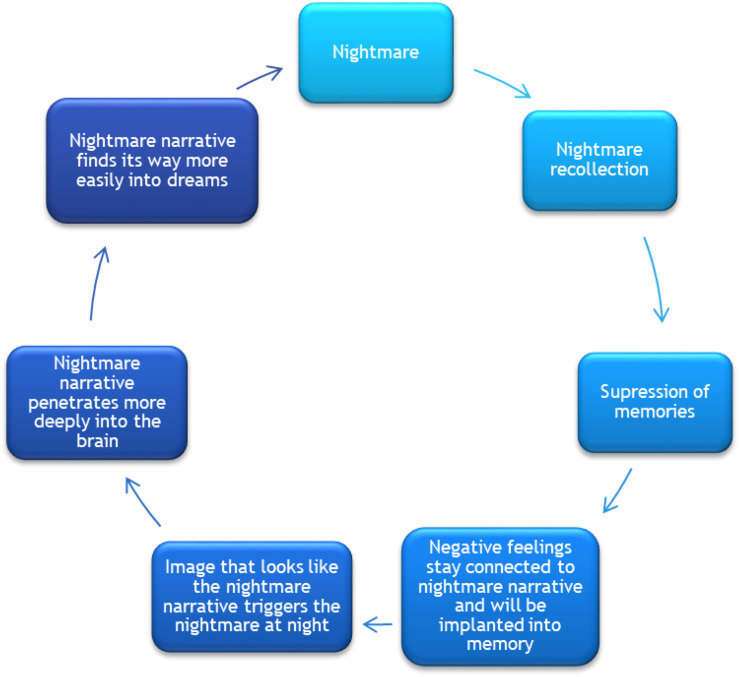
Vicious circle of nightmares. Adapted and translated from *Imaginatie-en rescriptingtherapie voor nachtmerries* (p. 15) by Van Schagen et al. (2012), Bohn Stafleu van Loghum. Copyright 2012, Bohn Stafleu van Loghum Publishing. Reprinted with permission.

This article focuses on the treatment of PTSD-related replicative nightmares. Replicative nightmares are dreams in which the traumatic event is re-experienced during sleep. These traumatic nightmares are one of the main PTSD symptoms (American Psychiatric Association, 2013). Nightmares lead to extra stress and anxiety on awakening and throughout the day. They can form an obstacle to daily functioning in that people avoid certain places, objects, and feelings in an attempt to suppress the fear and memory of the nightmare story. However, as explained in Figure 1, if memories of the nightmare are suppressed, negative feelings still remain connected to the nightmare, the narrative will be implanted deeper into memory and will more readily set off nighttime dreams (Van Schagen and Lancee, 2018).

### Imagery Rehearsal Therapy (IRT) of PTSD-Related Nightmares

Research shows effective results of IRT on traumatic nightmares, indicating that imagery rehearsal had large effects on nightmare frequency, sleep quality, and PTSD symptoms. Since IRT has received the most empirical support, we focus on the method of IRT (Casement and Swanson, 2012). IRT, a CBT technique, has three stages: *exposure*, *rescripting*, and *rehearsing*. *Exposure* involves recalling the nightmare and writing it down. The importance of rewriting the narrative can be declared from the “Emotional Processing Theory” (EPT; Rauch and Foa, 2006). This CBT theory points out PTSD is maintained by faulty perceptions whether environments or persons are safe (Rupp et al., 2017). *Rescripting* includes rewriting this dysfunctional interpretations and stopping the vicious circle of nightmares by changing the theme, story line, ending, or any part of the dream to a more positive and acceptable one. Ultimately, the rewritten dream scenario will be *rehearsed* so that patients can displace the unwanted content when the dream recurs. This involves having individuals found an active response to events that have disempowered and overwhelmed them in the past (Elbrecht, 2018; Hinz, 2020). In general, this technique is practiced for 10–20 min every day while awake (Germain, 2013). The psychotherapeutic IRT protocol as described by Van Schagen et al. (2012) prescribes seven sessions. Trust building is expected to occur in the previous sessions, before starting with exploring the traumatic nightmare memories. Positive attachment is essential to experience in recovering from traumatic events. The relationship between individual and therapist is the fundamental change agent in all psychotherapeutic methods, including art therapy (Malchiodi, 2020).

### Treatment Guidelines

Although IRT is recommended as a treatment of PTSD nightmares, art therapy might be promising because it works with non-verbal images and memories. Official guidelines for PTSD treatment (Foa et al., 2009) also presume that art therapy can be helpful in reducing depression and trauma-related symptoms such as alexithymia, dissociation, anxiety, nightmares, and sleep problems. Other positive results of art therapy mentioned in the guidelines are: increased emotional control, improved interpersonal relationships, and improved body image (Johnson et al., 2009; Schouten et al., 2018).

IRT combined with art therapy could be an interesting and effective combination. The current study investigates how PTSD-related nightmares can be treated using art therapy based on IRT. Useful interventions based on IRT and art therapy for treatment of PTSD-related nightmares are documented. The object is to contribute to the development of knowledge and interventions in the field of therapeutic trauma treatment and to establish a basis for research of the usefulness and effectiveness of this intervention.

## Materials and Methods

In this qualitative study we used data triangulation based on the combination and integration of systematic literature review and field research. The field research was conducted by interviewing professionals experienced in trauma treatment based on Imagery Rehearsal-based Therapy. The analysis of the in-depth interviews was performed based on the Grounded Theory Approach (GTA; Corbin and Strauss, 2008).

First, a systematic literature review was carried out to gain insight in the existing knowledge about IRT and art therapy as treatment for PTSD nightmares. To find matching results, the literature content had to include interventions based on IRT and art therapy-based interventions in the treatment of traumatized adults suffering from nightmares following PTSD (independent of type of trauma or type of trauma population). Keywords were: “imagery rehearsal” or “imagery rescript^∗^,” “posttrauma^∗^ nightmar^∗^” or “posttrauma^∗^ dream^∗^” or “posttrauma^∗^ memor^∗^” and “art (psycho)thera^∗^,” or “creative art therap^∗^,” or “expressive art therap^∗^.” Google Scholar, PUB MED, MEDLINE, Academic Search Complete, CINAHL Plus, Narcis, SAGE Navigator, and ScienceDirect were searched up to April 2019. The broad search resulted in 123 references. All studies on children and other forms of trauma therapy and techniques were excluded. After removing the duplicates and reviewing the titles for relevance, the list was reduced to 50 articles which were potentially relevant. Due to the small number of studies meeting the selection criteria, article references were studied in which additional useful studies were found.

The articles were studied in order to understand and apply existing IRT protocols in the treatment of traumatic nightmares. At the same time articles were studied to understand the use of art therapy in relation to traumatic nightmares. It was investigated whether there were similarities between the psychological guidelines described based on the protocols and the interventions described that are already used in the practice of art therapy. Based on this literature study, a framework was developed to inventarise the basic elements of IRT-based art therapy: the phase distribution, the interventions and the conditions (see Figure 3).

After the literature review, field research was performed. In-depth interviews were held to give input for and feedback from this framework, adding new content on the elements of phasing, interventions and preconditions in art therapy-based imagery rehearsal. In this iterative process, new interview data were compared with previous literature data and previous literature data were repeatedly compared with new interview data. Interview data were also compared with each other. The qualitative content analysis and the iterative process and constant comparison are key aspects of Grounded Theory methodology (Corbin and Strauss, 2008). We analyzed the interviews in phases of open, axial, and selective coding, which resulted in three main themes. The interviewed respondents were asked to read the summaries that emerged from the data analysis and to give feedback. This feedback confirmed the analysis. Based on the process of constant comparison we integrated the results of the systematic literature review and the results of the analysis of the interviews. Informed consent was obtained for using cases materials.

Respondents in the interviews were five trauma therapists with at least 25 years of experience: four art therapists and one psychotherapist. All therapists were employed at specialized trauma centers: mental health care institutions with post-trauma adults who suffered from having nightmares. This sample of therapists is chosen based on a very limited presence of these types of clinicians in practice and based on a network of specialized trauma centers. They included IRT intentionally, based on the integrated treatment in the specialized setting. The psychotherapist was a CBT-trained psychologist, not using art therapy interventions. The art therapists had no specific additional training in IRT/CBT.

The interview data included open, axial and selective coding in order to identify themes and patterns in the transcript interviews. All respondents signed the informed consent form.

## Results

### Literature Review of IRT and Art Therapy

Based on the literature review, preconditions for participation are described and useful art therapy elements are linked to the phases of IRT (exposing, rescripting, and rehearsing). The focus of effective PTSD treatment is to help patients break through experience avoidance and support new empowering narratives. Before patients participate in IRT, it is important to consider the patients’ mental and physical health state in general terms. Patients have to make sure they do not suffer in terms of experiential avoidance (Lobban, 2016). This term points out (un)conscious strategies to avoid an unpleasant experience, such as: substance abuse, eating disorders, or dissociation. When patients suffer from experiential avoidance strategies, they hold on to a state of mind in which they lose contact with the here and now in an attempt to deal with powerful emotions. If patients are not able to recover from their avoidance strategies, traumatic memories will occur in nightmares and flashbacks. This results in remaining symptoms (Schouten et al., 2018).

Useful art therapy elements in re-integrating the traumatic memory into the brain have been described. Various experts state that, in general, art therapy stimulates the link between the sensory and cognitive parts of the brain (Nanda et al., 2010; Chapman, 2014; Tripp, 2016; Elbrecht, 2018; Hinz, 2020). Art therapeutic assignments in trauma treatment focus on the part of the memory they are unaware of, and patients become aware by communicating and documenting images of traumatic memories and through rituals, symbols, and metaphors (Lobban, 2018; Hinz, 2020; Malchiodi, 2020). Therapeutic art interventions offer an opportunity to distance oneself from emotion, allowing for cognitive integration of emotion and stimulation of meaning-making processes (Hinz, 2020). Using metaphors such as line, form and color gives patients a safe, controllable and indirect way to find access to trauma-related content and prevent them from being overwhelmed (Johnson et al., 2009; Wise and Nash, 2013; Elbrecht, 2018).

**FIGURE 2:**
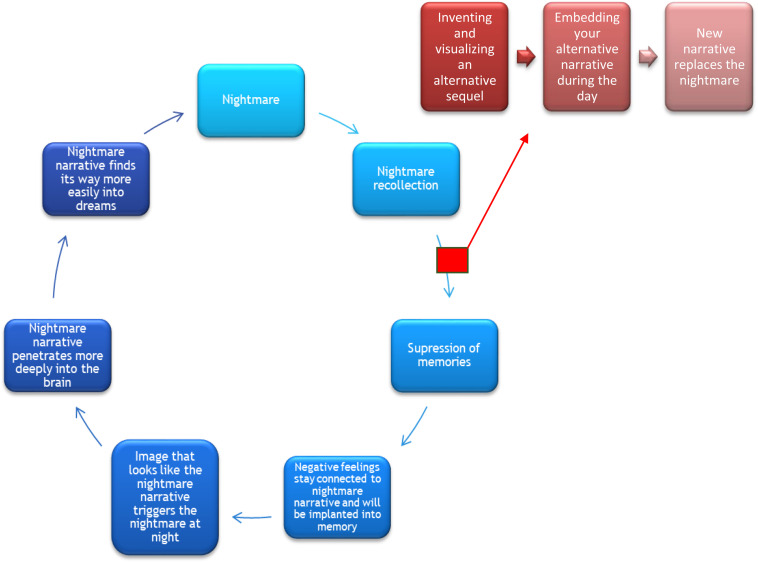
Stopping the vicious circle of nightmares. From *Nightmares in art therapy* by Van Tuin and Versluys (2019). Addition to Van Schagen et al. (2012). Bohn Stafleu van Loghum Publishing. Reprinted with persmission.

IRT is not only about recalling the nightmare memory, but also about changing the story line and implementing this new, empowering memory into one’s head (see Figure 2). Malchiodi (2020) describes how Gray (2015) states creating positive and imaginair rehearsing narratives are supporting and strength-based experiences that reinforce a sense of well-being to individuals. Art and creativity consist of imagination and can go beyond memories and reality (Johnson et al., 2009; Van der Kolk, 2014; Hinz, 2020). The phase of rescripting and rehearsing is supported by the Emotional Processing Theory (Rauch and Foa, 2006) and linked to art therapy research (Elbrecht, 2018; Hinz, 2020). In addition, art therapy treatment encourages humor, activity, playfulness and creativity. All of this is useful in changing memories and finding new perspectives (Johnson et al., 2009; Lobban et al., 2018). When implementing the new storyline into the brain, trauma-related nightmare memories can be adjusted, literally, in visual and tangible artworks. The artwork will be an image to hold on to in the process of replacing the traumatic memory image.

**FIGURE 3:**
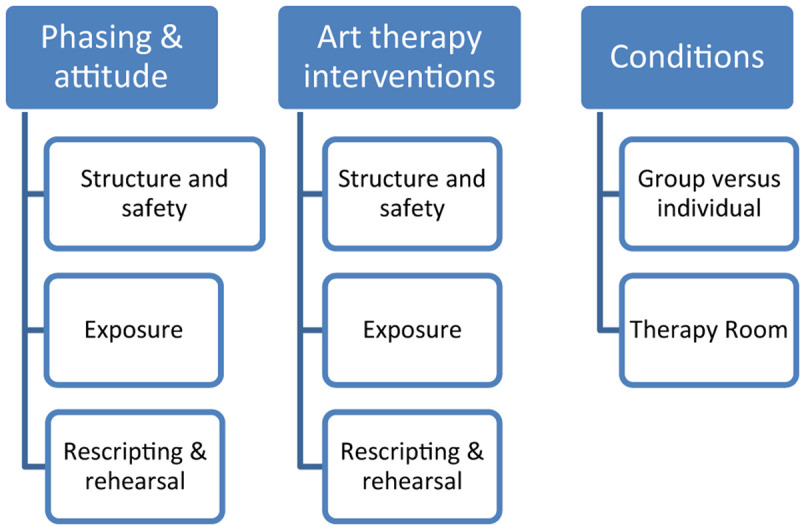
Framework of the basic elements of IRT-based art therapy.

### Integrated Results From Interviews and Literature

**FIGURE 4:**
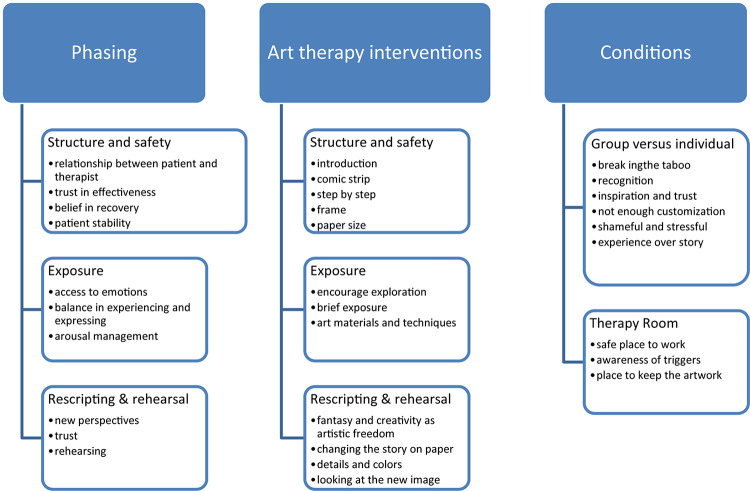
Major themes and content for imagery rehearsal-based art therapy.

Based on the integrated data, Figure 4 shows what content has been added to the framework of the basic elements of Imagery Rehearsal-based Art Therapy (Figure 3).

#### Theme 1: Phasing

##### Structure and Safety

The interview data identifies elements in ensuring a patient’s safety and relaxation. In the first place, the respondents emphasize the importance of a relationship of confidence between patient and therapist, the trust held by patients and therapists in the effectiveness of the therapy and the belief of the therapist in the patient’s capabilities for recovery.

They need to discover that …they can express their feelings, that it gives them something important. These are steps that it is necessary … for seriously traumatized people to take (respondent 2).

The need for a safe relationship can be explained by the understanding of “containment” (Finlay, 2015). Containment can be described as a process in which the therapist offers individuals a safe place to put in their thoughts, feelings and experiences. The therapist receives and represents those experiences, to make them easier to control and understand. This results in the ability to manage arousal.

In trauma focused art therapy, the art material is used to offer individuals a feeling of well-being and safety. The art objects are controllable and visual representations of what is experienced. Therefore, the process of containment not only takes place between therapist and client, but also between art material and client (Hinz, 2020; Malchiodi, 2020). Especially for those individuals who are suffering from (social) traumatic experiences, building a safe relationship with material, is essential to experience the feelings of trust and control (Lobban and Murphy, 2019).

##### Exposure

The second phase, in which exposure takes place, involves recalling the nightmare and writing it down. The respondents mention that, during exposure, patients must be able to access and explore emotions related to the nightmare story. Patients often experience high arousal when recalling nightmare stories and emotions of anxiety.

Re-experiencing a nightmare evokes a great deal of stress… exposure is real. You alternate between the emotion itself and working it out. You ask them to put everything they experience straight onto the paper, when stress levels are extremely high… in order to immediately give emotional value to their experiences (respondent 2).

The importance of the ability to manage arousal can be declared from the theory of “Window of tolerance” (Siegel, 2010). Trauma is experienced on top of the window, in state of hyper arousal: experiences are too big to understand. Under the window individuals experience a state of hypo arousal, in which they experience feelings of hopelessness and helplessness, dissociation, and unable to act (Corrigan et al., 2011). Within the marges of the window there is an optimal state of arousal: emotions are experienced as controllable and manageable to become nitrated and processed (Rotschild, 2014; Van der Kolk, 2014; Levine, 2015).

Interviewees stress the fact that it is necessary for patients to break through their resistance and avoidance in order to stop the vicious circle of the recurring nightmare (see Figure 1). They cite the importance of balancing between experiencing and expressing emotions during exposure. Moreover, the clients’ coping skills are decisive in managing patients’ arousal level and thus in gaining access to emotions. The respondents mention the importance of patients’ abilities to manage their own arousal, to avoid states of being overwhelmed or dissociating. The respondents proffer tools to help patients stay in the here and now, rather than shying away from their emotions. Tools that can be helpful include essential oils, gel packs, hot candies, a glass of water, stimulus mats, or balls, polished stones. The use of tools, even as the use of art materials as a way to manage arousal with an increasing sense of mastery and self-direction, is pointed out by several authors (Lobban, 2016; Schouten et al., 2018; Hinz, 2020; Malchiodi, 2020).

##### Rescripting and Rehearsal

Cognitive integration finds place at the end of the session (Malchiodi, 2020). Creating positive and imaginair rehearsing narratives are supporting and strength-based experiences that reinforce a sense of well-being and being “in control” to individuals (Mussche, 2014; Gray, 2015; Elbrecht, 2018). Rescripting offers individuals an active response to events that have disempowered and overwhelmed them in the past (Gray, 2015; Elbrecht, 2018; Hinz, 2020). According to the art therapists interviewed, the final phase of changing the story line of the nightmare into a more acceptable one for the patient takes place in art therapy by transferring the story into a rehearsed comic strip. Rescripting involves changing the theme, story line, ending, or any part of the nightmare into a more positive and acceptable one for the patient. The art therapists report how patients repeat their nightmare storyline in comic strip form, and how they change the story where it is most unpleasant and frightening. They point to the importance of discovering new perspectives on the story in order to create new storylines.

I think humor is important in art therapy, and so is calling on their fantasy and creativity so that they can find their own humorous solutions. … This puts them in a setting where they can feel free to play around a little …, which helps them find their way out of the deadly serious and immense anxiety (respondent 3).

The respondents emphasize how important it is that patients put forward new storyline ideas themselves to make sure the story becomes *their own story*, one that is fairly realistic and makes sense to them. The experience of “having an active response to that what has disempowered” is stated by several authors (Elbrecht, 2018; Hinz, 2020; Malchiodi, 2020). The rewritten nightmare scenario must be rehearsed to displace the unwanted content, the art materials are thereby able to support a new sense of purpose, grip, planning and flexibility (Hass-Cohen et al., 2018). Cognitive integration is accomplished by letting patients imagine the new storyline or look at the new image, and practice it when the nightmare recurs (Germain, 2013).

#### Theme 2: Art Therapy Interventions

During the interviews, art therapists were asked for concrete art therapy interventions they used, ones that might help create structure and safety, explore emotions or rewrite the nightmare story. The art therapists created and used their own interventions in their practice, and they were not necessarily consistent with each other. Structure and safety are created by the following aspects.

##### Structure and Safety

Interviewees point out the importance of a structured attitude on the part of the therapist in working with PTSD patients. Interview data show that therapists introduce their method to patients by explaining structure and rationale of the art therapy nightmare treatment to make sure they are prepared. On the one hand, an explanation makes the patient more willing to participate, and on the other hand, it keeps the arousal level manageable (Window of Tolerance; Siegel, 2010). An intervention that provides structure and safety to patients is working with comic strip assignments. This allows patients to explore the nightmare step by step, image after image, using the structure of a sheet of paper with pre-drawn frames of an average size. Based on the clinician experience, the numbers of comic strip images needed to explore the nightmare and the corresponding emotions varies from 4 to 7 images.

The fact that you have a sheet of paper with a beginning and an end, generally with 5–7 frames…. I prepare and fold the sheet ahead of time, so that they see it taking place. This provides a great deal of structure (respondent 2).

The theory behind symbolism and metaphor can support the use of images in art therapy in providing structure and safety. Hinz (2020) describes the language of metaphors offers patients a way to control their expression. Symbols can indirectly communicate a story; they are visual but not communicated by verbal language yet. This allows patients to explore step by step, in line with their window of tolerance, to support integrated processing (Collie et al., 2006; Schouten et al., 2018).

##### Exposure

Exposure is about recalling nightmare stories and re-experiencing emotions. The Emotional Processing Theory (Rauch and Foa, 2006) emphasize therapist to encourage patients to explore their nightmare stories. Sample questions are: “Where were you?,” “Who was there with you?,” “What did your surroundings look like?,” “What sounds did you hear?” could be asked to encourage patients to express their imagination in vivid terms. “What does it look like?,” “What elements need to be bigger, what ones should be smaller?,” “How do you feel, what do you smell?,” “What colors go with this emotion?,” “Is the environment brightly lit or dark?.” By asking these questions, the therapist becomes a side-viewer in the traumatic memories of the individual (Hinz, 2020; Malchiodi, 2020). The therapist functions as a save and supportive place to individuals express their experiences (Containment; Finlay, 2015).

When someone tells you about this, you need to ask questions …what kind of road was it. is it winding…is it straight…um.how can you indicate that it is suspenseful, gripping…is that red.is that black?.is that dark.is that light?. How else can you indicate the anxiety you are feeling? Look at the colors you have here in your palette, what color is best suited…? (respondent 2).

Art therapy has been described as helpful in overcoming blockages to gain access to implicit traumatic memories (Nanda et al., 2010; Chapman, 2014; Tripp, 2016; Elbrecht, 2018; Lobban and Murphy, 2018; Schouten et al., 2018; Hinz, 2020). Art therapeutic assignments in trauma treatment focus on the part of the memory they are unaware of, and patients become aware by communicating and documenting images of traumatic memories and the use of material, rituals, symbols, and metaphors (Lobban, 2018; Hinz, 2020; Malchiodi, 2020).

In art therapeutic practice, the duration of an art therapeutic IRT nightmare treatment appears to vary. The respondents in this study say that the exposure must be completed within one or two sessions, in order to frame the arousal level. Malchiodi (2020) mentions that the actual duration of trauma intervention is variable, each individual responses different to building a relationship of trust, due to what happened to them. Individuals also vary in the capacity for imagination and cognitive integration. One therapist explains that, if patients exhibit an exceptionally high arousal level that has a negative influence on the exposure, she has them experience only a brief exposure. In such a case, she asks patients to start out by evoking and working out an image of the ending they want for their nightmare rather than leading them through the frightening nightmare story. Once the desired ending has been sufficiently defined, they work back in phases to the traumatic nightmare story. This ultimately results in a manageable level of arousal (Siegel, 2010) and integration of the new story and the nightmare story (Malchiodi, 2020).

When it comes to useful art materials and techniques, some therapists emphasize the value of colors and blending techniques for evoking patients’ emotions, while others emphasize the value of expressing the story in simple drawings or photo collages. They also point out that art materials and techniques should be user friendly (easy-to-use) and not too hard to work with. If materials are too hard to deal with, they say, it can cause patients extra stress (hyper arousal). Examples of material that is harder to work with is wood, metal, stone or more technical artistic techniques. The concrete examples of easy-to-use art materials, the respondents work with, vary from pastel crayons, drawing pencils and watercolor pencils and paint to images and texts from magazines.

In my opinion, pencils act too much as a control material, which makes it hard to show emotion. They have an easier time of it if they use watercolors. … I like to work with acrylic paints, but they are too time-consuming; if people have little experience with them. they have a hard time completing a drawing by the end of the session (respondent 2).

The respondents seem to choose materials based on their assumptions about structure, practical use and expressive possibilities, based on their experience and theoretical backgrounds. These choices also are guided by their own and their patients’ preferences.

Several authors (Elbrecht, 2018; Jones, 2018; Hinz, 2020; Malchiodi, 2020) point out the variety of art materials and their character. All authors emphasize the function materials could have in deciding whether they support the aim of the art therapy session. For example, watercolors evoke emotions rapidly, where crayons provide intensity and strength and collage material provide structure (Elbrecht, 2018; Hinz, 2020).

##### Rescripting and Rehearsal

According to the interviews, the final phase of changing the story line of the dream into a more acceptable one for the patient is accomplished by transferring the story into a comic strip. Therapists show how patients repeat their nightmare storyline in a comic strip, changing the story where it is most unpleasant and frightening.

You have become a victim … Your assignment is … to fashion the nightmare into a story again, but … so that in the end, you are the winner and no longer the victim (respondent 1).

The respondents as well as the literature mentions that the therapists can encourage patients to find alternatives to the story. The visual character of the artwork offers patients distance and the ability to reflect and conceptualize new narratives (Elbrecht, 2018; Hass-Cohen et al., 2018; Hinz, 2020). According to the interviews, important rescripting elements are: patients are permitted to use all their fantasy and creativity as a form of artistic freedom and to change the story on paper. “What can be perceived in an image can perhaps be more easily managed in real life” (Hinz, 2020, p. 80).

Malchiodi (2020) points out important elements Gil (2017) observed as dynamic and empowering narrative aspects. Those elements include affects become available, interactions become varied, new elements are added, different locations occur, new objects are included, narrative outcomes differ, and more adaptive responses emerge. One of the participating therapists in this study brought up the example of a traumatized woman who was able to capture her attacker in her new storyline. By changing her story in this way, she experienced control over her traumatic story.

And then they try to encourage you by saying, this is about you and your emotional world. You suffer from it; you want to move on. This has nothing to do with your mother or your family; they’re not here now. You do this for yourself, using this technique, and you do them absolutely no harm (respondent 1).

Important elements of art therapy that contribute to rehearsal have proved to be working on the new visual story on a detailed level, using vivid colors, and repeatedly looking at the new image, the storyline newly created in this new version. Rehearsing is necessary in adapting the new (visual) written narrative (explicit memory) into the consciousness (explicit memory) (Elbrecht, 2018; Malchiodi, 2020). Therapists suggest patients to take a photo on their phone or pin the artwork narrative to the wall.

Make sure to use plenty of detail in the final frame: color it or paint it, just so it shows up. … And after that I often ask then to work out their entire story in greater detail. So, if you haven’t colored it yet, color it now, […] and develop as much detail as you can. Taking a longer look, considering it, thinking about it, it all helps to implant the new story (respondent 3).

The following case vignettes are patients whom the interviewed therapists worked with. This therapy took place in a specialized trauma center, in a broader treatment program consisting of mostly verbal therapies.

#### Case Vignet 1

**FIGURE 5:**
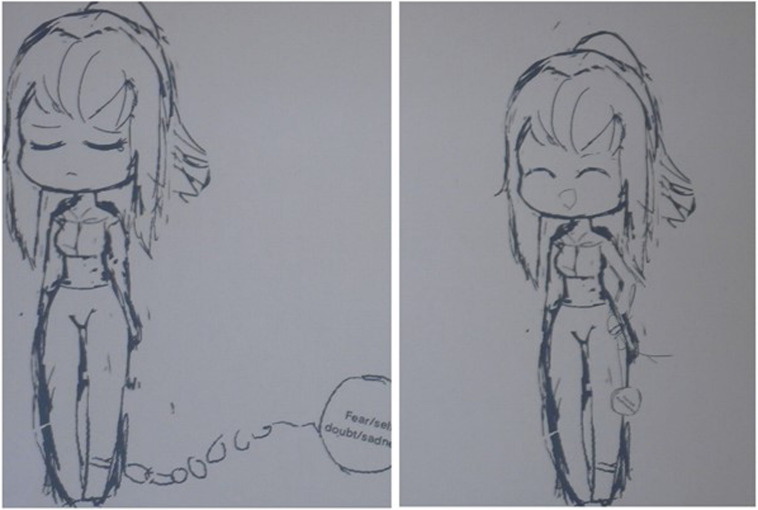
The drawing of the “intangible feeling of fear” symbolized in a heavy ball on the chain on her leg and the drawing in which she transformed it into a tennis ball.

“Alicia,” an 11-year-old girl with PDD-NOS, suffers from nightmares caused by an “intangible feeling of fear.” She has converted this fear into the first drawing (see Figure 5). Drawing and looking at her fear puts her in touch with her feelings. This stage is called exposure; a way to express painful experiences (Malchiodi, 2020). In the following image (Figure 5), rescripting has been applied to the exposure drawing. The heavy ball on the chain on her leg has now been transformed into a tennis ball. This scripted image, also known as the “Happy ending,” shows the future image of her fear; the ball is now so small that the girl can hold it. Consciously looking at and permeating the “Happy Ending” can evoke a feeling of reassurance and confidence. This is in line with Gray’s (2015) statement; creating positive and imaginair rehearsing narratives are supporting and empowering to a sense of well-being. The IRT is practiced with the vision of the future: by regularly viewing it, this overwrites the image of fear. Because the identity has been rewritten from the inside, the implicit memory, in a way the individual is unfamiliar with, the new imagined narrative needs time and practice to adjust to the explicit memory, the consciousness (Elbrecht, 2018).

#### Case Vignet 2

**FIGURE 6:**
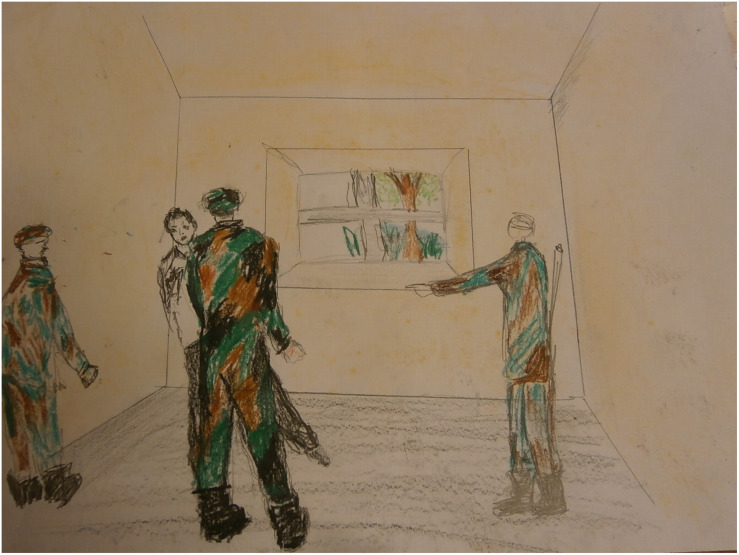
Drawing of traumatic experiences around a military checkpoint.

**FIGURE 7:**
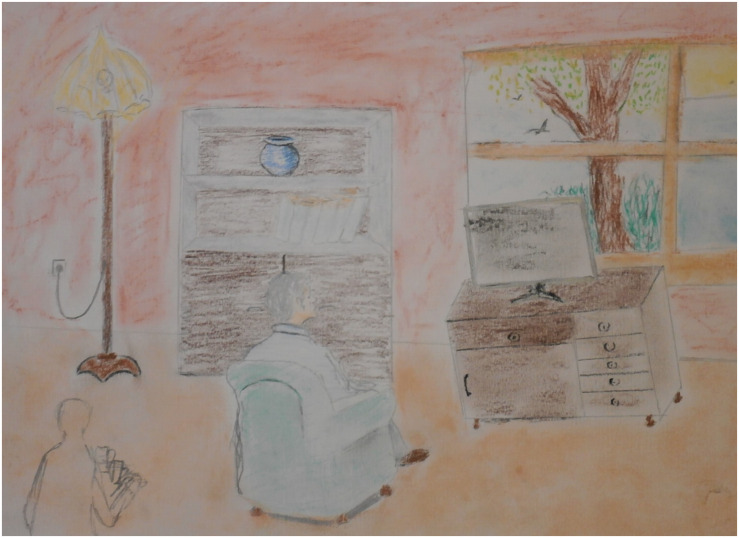
Rearranging the room.

**FIGURE 8:**
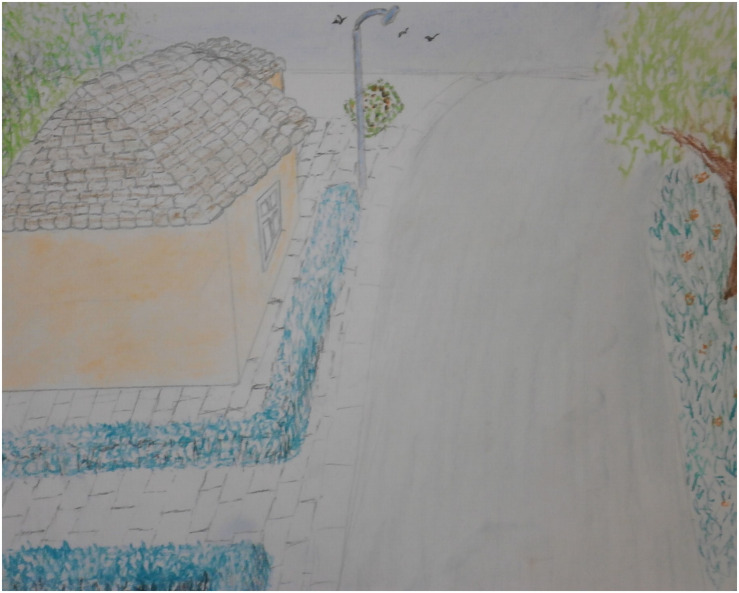
Zooming out of the room.

“Omar,” a 35-year-old man from Iraq, is being treated for his nightmares and traumatic experiences around a military checkpoint. His nightmares hardly decreased in severity and frequency with psychological IRT treatment. He drew the checkpoint in art therapy. At first, he drew one soldier, who caused the threat in the room. Later the man made the drawing more realistic; he was threatened by three soldiers (see Figure 6). This addition to the scene created a more intense experience of exposure; the intensified threat in the image made sure that the man could really feel the fear in question. In the art therapy session, rescripting was applied with a focus on the threatening room by “rearranging” the room and by zooming out (see Figures 7, 8). Instead of focusing on the repetitive narrative, new narratives were explored. With the help of the artwork the man could perceive the room from a larger distance and play with the experienced atmosphere. The frightening associations with the room were replaced by pleasant experiences. This new narrative creates a new sense of meaning making, purpose and cognitive integration (Hinz, 2020; Malchiodi, 2020).

#### Theme 3: Conditions

##### Indications: Group vs. Individual

The respondents brought up arguments to consider a group vs. an individual therapy setting. They worked with IRT-AT with both individuals and groups and mentioned advantages and disadvantages. This concerns safety, and what one needs to gain benefit from the therapy. Porges (2012) and Lobban (2018) point out group therapy elements which support healthy social interpersonal relationships. Those include: breaking through the taboo and discussing painful memories, acknowledging stories of suffering, encourage one another to find new narratives and help one another to trust in the recovering process. This is acknowledged by interviewed therapist.

After a person has reworked their trauma three times, someone says: “That nightmare has just gone plain,” … this can have inspiring and encouraging effects (respondent 3).

However, arguments against working as a group is that the art therapist cannot offer sufficient individual assistance in the form of coaching and discussing delicate and intimate themes. Sometimes themes can be shameful and stressful for patients; particularly when working in a group, they may never be forced to share their vulnerable, intimate themes. In art therapy, a compromise can be found by removing the focus on the trauma story (verbal narrative) and shifting it to the sensorimotor and emotional experience of creating the new story or artwork (Elbrecht, 2018; Hinz, 2020).

##### Therapy Room

Traumatized patients must be able to work in a safe space and the therapist must be aware of triggering factors such as noises, objects, and smells that can evoke memories of the trauma in the patient. Malchiodi (2020) describes how individuals differ in their perceived threats, consisting of triggers caused by the environment, social surrounding, materials, and activity.

Especially if we are talking about people who have lived in a circumstance of domestic violence or sexual abuse, an initial step is to remove all triggers from the room (respondent 5).

Multiple authors emphasize the need of a calm a focused atmosphere where patients feel safe enough (Van der Kolk, 2014; Elbrecht, 2018; Hinz, 2020; Malchiodi, 2020). This is in line with offering the individual a therapy within his or her window of tolerance, which is essential to manage the level of arousal and overcoming traumatic experiences (Siegel, 2010; Levine, 2015). Supporting to a safe therapy environment is to store artworks safely. This can be accomplished by giving each patient his or her own folder and putting away each folder securely locked. The storage can be seen as a ritual to create a safe place for and offer distance from the traumatic memories (Malchiodi, 2020).

All patients have folders that contain their work …. The work cannot fall out, no one will see it, it is safely locked away and no one can touch it (respondent 1).

## Discussion

In this article we investigated and documented useful practice-based art therapy interventions in the imagery rehearsal treatment of trauma-related nightmares. IRT is effective for PTSD-related nightmares, but it proves to be very challenging for patients to find access to their traumatic memories, because these memories are saved in non-verbal, audio and/or audiovisual language. In our study, art therapy emerges as an additional, possibly more effective angle for treatment, especially for patients who benefit least from verbal exposure techniques. This is because art therapy addresses images rather than words. It offers an experiential, non-verbal entry point which can help to find access to traumatic memories for the treatment of PTSD-related nightmares. This can provide treatment for traumatized patients, especially for those who are unable to talk about traumatic memories and unable to tolerate verbal exposure treatments. Based on our investigation, the use of IRT techniques combined with the advantages of art therapy might well be more effective for patients suffering from trauma-related nightmares than IRT alone.

On the basis of the literature review and the interviews, theoretical and practice-based knowledge was inventarised and integrated into an overview of the contents of important elements, divided into three themes: phasing and attitude, art therapy interventions and conditions. Each of these themes is described in relation to the phases of imagery rehearsal treatment: (1) structure and safety, (2) exposure, and (3) rescripting and rehearsal. The added value of using art therapy with IRT is that patients are able to gain access to traumatic experiences, to experience feelings and to break through avoidance. Exposure and re-scripting take place in art therapy in a more indirect, experiential and sometimes playful manner. Thanks to this, avoidance may be less frequent and can sometimes be broken through. The story of the trauma-related nightmare can be studied more closely, experienced, drawn and rewritten in the artwork, while imagination, play and fantasy provide creative space.

This is also found in the literature. Research has shown that, when working with trauma exposure, art therapy provides added value through aspects such as living through emotions and breaking down avoidance because it is a form of non-verbal therapy, and because frightening memories are concretely defined in artworks (Litz et al., 2009; Hinz, 2020). In addition, during the rescripting phase, the non-verbal angle helps in gaining new perspectives and taking distance from memories of nightmares (Van der Kolk, 2014). This increases the scope for change, and there are indications that working intensively with non-verbal means such as symbols and colors makes it easier to implant new memories (Johnson et al., 2009).

Although this study was carried out with the greatest possible accuracy, it also has limitations. First and foremost is the small number of respondents interviewed. Such a small sample implies that the research conclusions are not necessarily applicable to all therapists, and so they must be used with some caution. However, the findings in relation to the combination of IRT and art therapy do give clear indications for Imagery Rehearsal-based Art Therapy (IR-AT) for patients with trauma-related nightmares. It can also be commented that the statements of almost every respondent are supported by findings in the literature. These findings give the practice-based findings a broader base. A second limitation of this study is that no patients were interviewed, and so their perspective may not have come forward adequately. However, for the object of this study, inventarising effective elements, it was firstly important to gain more insight into the interventions used and to ask therapists about their manner of working. A third limitation is that more studies may well be available about art and/or IRT treatments for traumatic nightmares which were not included in this study.

The strength of this study is that the framework of orderly, collated aspects, and elements from this study (Figure 1) provides a clear starting point for tailoring IR-AT interventions for patients who suffer from trauma-related nightmares. For this purpose, we used available literature and practice-based knowledge of experienced (art) therapists to substantiate the practical art therapy interventions. The practical interventions identified can be traced back to the general IRT guidelines for treating trauma-related nightmares.

The description in this study of IR-AT offers a basis for further research. The field of art therapy stands to benefit from further research of Imagery Rehearsal-based Art Therapy for trauma-related nightmares. This could include, for example, research of (multiple) case studies or impact studies in which IRT is compared with Imagery Rehearsal-based Art Therapy for trauma-related nightmares. For subsequent research, it is also recommended to devote attention to increasing the size of the research population, including both art therapists in the professional field and experiences and opinions of patients. This will lay a solid basis for a better substantiation and optimal enlistment of art therapy for patients suffering from trauma-related nightmares.

## Data Availability Statement

The raw data supporting the conclusions of this article will be made available by the authors, without undue reservation, to any qualified researcher.

## Ethics Statement

Ethical review and approval was not required for the study on human participants in accordance with the local legislation and institutional requirements. The patients/participants provided their written informed consent to participate in this study. Written informed consent was obtained from the individual(s) for the publication of any potentially identifiable images or data included in this article.

## Author Contributions

SH and MS contributed to conception and design of the study and wrote sections of the manuscript. MS organized the data and wrote the first draft of the manuscript. Both authors contributed to manuscript revision, read, and approved the submitted version.

## Conflict of Interest

The authors declare that the research was conducted in the absence of any commercial or financial relationships that could be construed as a potential conflict of interest.
